# The impacts of family physician plan and health transformation plan on hospitalization rates in Iran: an interrupted time series

**DOI:** 10.1186/s12913-021-06685-w

**Published:** 2021-07-05

**Authors:** Samad Rouhani, Reza Esmaeili, Jamshid Yazdani Charati, Masoud Khandehroo

**Affiliations:** 1 Department of Public Health, School of Health, Mazandaran University of Medical Sciences, Sari, Iran; 2 Department of Public Health, School of Health, Social Development and Health Promotion Research Center, Gonabad University of Medical Sciences, Gonabad, Iran; 3 Department of Biostatistics, School of Health, Mazandaran University of Medical Sciences, Sari, Iran

**Keywords:** Health reform, Family medicine, Referral system, Hospital admission

## Abstract

**Background:**

Low and middle income countries has recently implemented various reforms toward Universal Health Coverage (UHC). This study aims to assess the impact of Family Physician Plan (FPP) and Health Transformation Plan (HTP) on hospitalization rate in Iran.

**Methods:**

We conducted an Interrupted Time Series (ITS) design. The data was monthly hospitalization of Mazandaran province over a period of 7 years. Segmented regression analysis was applied in R version 3.6.1.

**Results:**

A decreasing trend by − 0.056 for every month was found after implementation of Family Physician Plan, but this was not significant. Significant level change was appeared at the beginning of Health Transformation Plan and average of hospitalization rate increased by 1.04 (*P* < 0.001). Also hospitalization trend increased significantly nearly 0.09 every month in period after Health Transformation Plan (*P* < 0.001).

**Conclusions:**

Family physician created a decreasing trend for hospitalization in urban area of Mazandaran province in Iran. HTP with lower user fee in governmental public hospitals and clinics as well as fee-for-service mechanisms, stimulated both level and trend changes in hospital admissions. Some integrated health policy is required to optimize the implementation of diverse simultaneous reforms in low and middle-income countries.

## Background

Universal Health Coverage (UHC) has become a key national aspiration in designing and implementing of health policies [1]. Iranian health system faced with critical challenges toward UHC over 4 last decades e.g. poor referral system, high rate of noncommunicable diseases and health service provision with social protection. Many initiatives have been implemented to address these challenges [2, 3]. Strengthening Primary Health Care (PHC) was experienced in various models such as family physician plans as the main strategy in process moving toward UHC in Iran and around the world [2, 4, 5]. Powerful evidence demonstrated that quality improvement in PHC and referral system reduces secondary care admission and cost [6, 7]. Three dimension of UHC (extend to non-covered population, reduce cost sharing and including other services) was aimed in the last health reform in Iran known as HTP for improving service provision with low cost sharing [8]. Health economic literature stated that expanding service coverage with low cost sharing may stimulate service utilization and even moral hazards [9]. So both of recent health reforms in Iran had potentials to effect on hospital services utilization. Hospitalization rate has been used frequently for evaluating the impacts of policy reforms [10, 11]. This study aimed to assess the impacts of two recent health reforms on hospitalization rate in Iran. Comparative evaluation contribute to make judge and decision for rational and ethical resource allocation.

## Recent primary health care reforms in Iran

After Islamic Republic of Iran’s Revolution in 1979, Regional Health Network as a network of primary health care facilities expanded thorough the country in order to improve the health of population with a priority devoted to rural and unprivileged areas. Based on national and international assessments, the achievement was satisfactory, but it has been felt that more space is there to strengthen its PHC network [2, 3].

Iranian health system faced some critical challenges including poor referral system and high rate of non-communicable diseases. In response to these challenges, FPP was implemented in rural area in 2005 [2]. Then, development of FPP to the urban area began in 2013 in two provinces (Mazandaran and Fars). In these reforms a general practitioner (GP) as family physician was contracted to serve an enlisted population and is paid on capitation basis. The services include a basic package of primary health care that formerly was provided by state employed physicians and related staffs of community health centers. In rural area all physicians are based in the community health centers but in urban area some private offices are in contract as well. Enrolled population in rural area require to pay up to 30% of tariffs when they use services through their family physicians (e.g. 5000 Rials for doctor visit, 15% for laboratory and up to 30% for drugs) [12], but in urban area such services is almost free of charge [13]. This difference in copayments between rural and urban populations exist because by government subsidy rural population without buying any insurance premium could enroll to the GP but in urban area just people already having different sort of health insurance plan could be covered. Patients from both groups could benefit from inpatient and outpatient specialist services of public hospitals if they carry an issued referral letter from their family physicians. Family physicians could refer up to 10% of their patients for secondary care. In addition there are some other limitations such as items of drugs in each prescription, request for laboratory tests or images and so on. 80% of FP contract is paid monthly and the remaining 20% is subject to the results of seasonal assessment of their performance done jointly by staff of health insurance organization and district health authority [13].

## Health transformation plan in Iran

After introduction of FPP in Iran, Health Transformation Plan (HTP), with different objectives including facilitated secondary care at governmental public hospitals, reduction in out-of-pocket payments (co-insurance rates in public hospitals and specialty clinic), better quality of hospital services and so on was implemented at country level in May 2014 [14]. In this reform medical tariffs at governmental public hospitals were heavily (three to more than five times) increased, but attendees were charged between 3 to 6% of their hospital bills whether they carry a referral letter or not. The justification for such reform was mainly to create incentive for both providers in particular physicians by sharing generated incomes with them and patients by subsidizing their bills by government allocated resources. High public resources (from government and insurer organizations) allocated to support the HTP [3, 8].

## Methods

### Study design

This was a quasi-experimental design using Interrupted Time Series (ITS). ITS is the strongest quasi-experimental design for evaluating the impacts of health policies and reforms and does not require to comparison group [15, 16]. Several study designs are available for ITS studies [17]. In this study, outcome measure for the time series analysis was hospitalization rate across monthly intervals. As both reforms could have impacts on hospitalization rate, therefore the change on this indicator will be traced on three segments including the period before FPP, from the start of FPP to before the introduction of HTP, and the period after implementation of HTP.

### Data sources

Mazandaran is a province located in the north of Iran with 3,283,582 population (annual growth: 1.33). 50.37% of Mazandaran population are male and the median age is 33 year. 57.8% of the population live in urban area. This province had 48 hospitals, including 6321 beds in 2016 [18]. In this study we used data on insured people by Iran Health Insurance Organization (IHIO) in Mazandaran as the main financing agent for these two reforms. From provincial office of IHIO including the number of insured people and the number of inpatient records (Table 1). The study observations were determined as monthly hospitalization rate from March 2010 to February 2017.

**Table 1:** Population and hospitalizations in the Mazandaran province during the study period

Parameter	2011	2012	2013	2014	2015	2016	2017
Population covered by Iranian Health Insurance	1,628,919	1,682,767	1,708,193	1,577,596	1,674,328	1,676,317	1,432,290
Total number of hospitalization	126,862	129,861	124,561	132,715	152,478	155,358	151,698
Monthly average of hospitalization,	10,571	10,821	10,380	11,059	12,706	12,946	12,641

### Data analysis

A linear regression model is specified to explain study hypothesizes as following equation.

$$ {Y}_t={\beta}_0+{\beta}_1 time+{\beta}_2 interruption\ 1\ (FFP)+{\beta}_3 time\ after\ interruption\ 1+{\beta}_4 interruption\ 2\ (HTP)+{\beta}_5 time\ after\ interruption\ 2\ {(HTP)}_{+}e $$

In this model:

- Yt is hospitalization rate *(total monthly inpatient records divide on insured population)*
- *β*_*0*_ is the baseline level of hospitalization rate at the beginning of the time series
- *β*_*1*_ is the pre-intervention slope prior to FFP
- *β*_*2*_ is the change in level immediately after the FFP
- *β*_*3*_is the change in the slope from pre to post FFP
- *β*_*4*_ is the change in level immediately after the HTP
- *β*_*5*_ is the change in the slope from pre to post HTP
- *e* represents the value of residuals.

We used segmented analysis for estimating both immediate (level) and long-term (trend) impacts of interventions. It take ideally at least 12 observation points for each period to reduce seasonality effect [19]. Our sample size was 84 observation points, 19 observations before first interruption (FFP), 19 observations between two interruptions and 48 observation points after second interruption (HTP).

We conducted several diagnostic assessments. To detect autocorrelation between residuals we used Durbin Watson test which is corrected using the Praise-Winsten method [20]. We also used Augmented-Dickey-Fuller statistic to determine if the series was stationary. We estimated the Kolmogorov–Smirnov statistic to check the normality of the residuals. Bartlett test was used to assess the homogeneity of variance between residuals. All analyses were done in R version 3.6.1.

## Results

Regression model results are presented in Table 2. The average value of hospitalization rate was 6.32 per 1000 insured people at the beginning of study period. Inpatient rate appeared to increase 0.01 every month prior to the FPP (baseline trend). The findings in Table 2 indicate an increase in the level of hospitalization rate at beginning of FPP, but this was not significant. After the implementation of FPP, the hospitalization was replaced by a decreasing trend of − 0.056 for every month.

**Table 2:** Parameter estimates from the segmented regression in two interrupted model

Parameter	Coefficients	Standard Errors	t-statistic	Confidence Interval		*P* Value
**Initial level**	6.321	0.265	23.823	6.851	5.790	0.000
**Initial Trend**	0.011	0.024	0.473	0.060	−0.037	0.637
**Change in level after FPP**^**a**^	0.085	0.348	0.244	0.781	−0.611	0.807
**Change in trend after FPP**	−0.056	0.033	−1.679	0.067	−0.067	0.097
**Change in level after HTP**^**b**^	1.045	0.284	3.668	1.615	0.475	0.000
**Change in trend after HTP**	0.088	0.023	3.777	0.134	0.041	0.000

^a^FPP Family Physician Plan

^b^HTP Health Transformation Plan

**Fig. 1:**
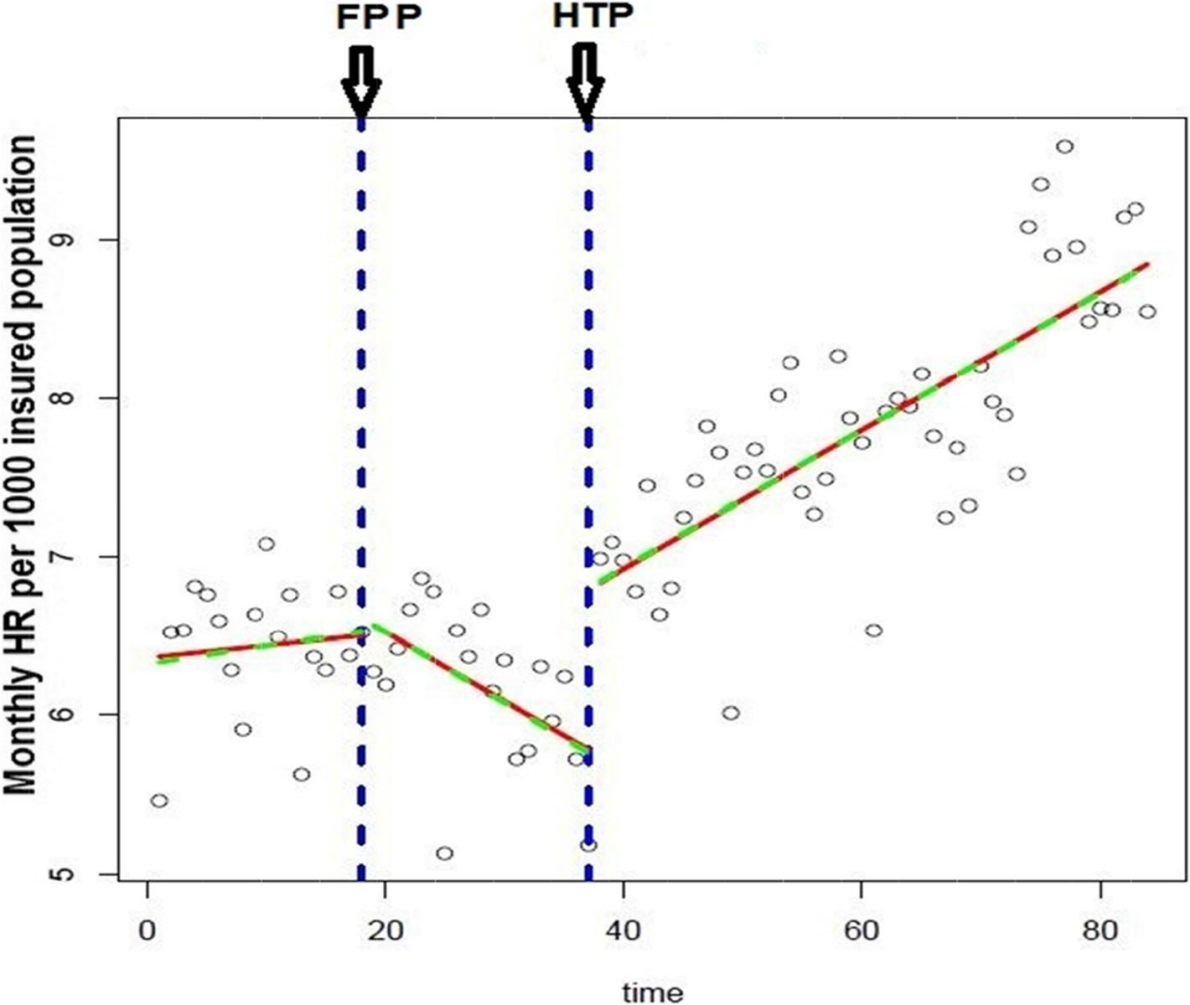
Segmented regression model of hospitalization rate with two interruption

A significant level change was appeared at the beginning of HTP and average of hospitalization rate increased by 1.04. Also a significant increasing trend was found for period after HTP that is nearly 0.09 change in hospitalization rate per month. Figure 1 shows the impact of both FPP and HTP on hospitalization rate in the study period.

## Discussion

We applied segmented regression in interrupted time series design to assess the impact of two major reforms in Iranian health systems on hospitalization rate. Segmented regression was recommended as a practical approach for assessing the impact of health policy change in low and middle-income countries [21]. In recent years various studies used segmented regression to assess the impact of different health policies on drug prescribing [22], drug utilization [23], hospital quality measures [24], maternal and child care [25], outpatient care [26], and inpatient care [11] in different counties.

The same with expected impacts of provider-continuity polices [27], the results indicated a decreasing trend of hospitalization rate after the implementation of FPP. These findings are different to the result of a study about the effect of FPP on hospitalization in rural area in Iran [11]. Rashidian, et al. reported that FPP in Iranian rural area has led to a significant increase of hospitalization rate. Rashidian, et al. have mentioned “access effect” and have argued that, unmet need in the rural area out-weighted the effect of FPP on hospitalization. Yet, after a decade, the improvement of health care accessibility in Iranian rural area and also different sets of public and private health facilities in urban area in Iran omitted the “access effect” of urban FPP and lead to decrease in the trend of hospitalization rate. In other words, the implementation of FPP in two different contexts resulted in different impacts on hospitalization rate. Another study in the United-States also confirmed access to effective primary care result in lowering the rate of hospital admissions [28].

After 9 years from beginning of rural FPP and 19 months from urban FPP, third reform known as Health Transformation Plan (HTP) was started in Iran [2]. Based on HTP components, it was expected that, the hospitalization rate may be affected after HTP. Therefor we entered HTP as second interruption in the analysis and the results demonstrated that implementation of HTP lead to significant changes in the terms of level and trend in hospitalization rate. In similar study in Fars province, Bayati, et al. [29] reported an insignificant increase of hospitalization service after HTP. Piroozi, et al. [26] also assessed the impact of HTP on specialist outpatient visit rate in Kurdistan province in Iran and have reported an increased rate for outpatients visit after beginning of HTP. Since that, outpatient service and inpatient care are considered as complementary goods rather than substitute services in health care markets [30], an increased level and trend of outpatients specialty visits have stimulated inpatient services after HTP in Iran. Furthermore, lower co-insurance in governmental public hospitals and clinics as well as fee-for-services payment mechanisms have increased inpatient care utilization that could be a sign of provider^,^ s induced demand or consumer^,^ s moral hazards [3]. Toward significant role of FPP in referral system and try to approximate rational provisions of secondary care in Iran, pitfalls in services process should be focused. by current policies of two levels of primary and seconndary health services in this province, GPs at PHC level as capitated gatekeepers would not have enough incentive to stop their patients for going to secondary care. But specialists at secnodery level when are paid based on volume of care would have enough incentives to accept more patients even for the services that could be effectively deliverd by their family physicians or over supply the services to the patients i.e. more hospitalise the patients if there is not adequate patients coming to them. In other words when there is no pain for gatekeeprs for more than neccsarry use of secondary care by their enroled population, but there is adequate rewards for care providers at secondary level to provide more services, induced demand at secondary level is expected. In consumer side when they could have access to nearly free hospital services, their brake against hospital charges would not work appropriately providing more opportunity for over supply of servicess at secondary level to them. This is evidently an misapproprite mechnism of creating incentive for different parties to provde right care to right patints.

In brief, the results of this study demonstrated different impacts of FPP and HTP on secondary care utilization in Iran. FPs as gatekeepers by filtering attendees reduced inpatient care utilization through more logical referral pattern, but the increasing impacts of HTP on hospitalization would be controversial, from addressing unmet needs to the stimulating moral hazards. Considering the implementation of family physician in both rural and urban areas of Mazandaran province before HTP, the impact of HTP on hospitalization rate might be different in other provinces without family physician in urban area. Based on the findings of this study, it is necessary that policy makers review the undesired impacts of two reforms and arrange adequate incentives or disincentives among different parties i.e. by introducing GP fund-holding for both better linkage of service providers and better utilization of limited resources for the benefit of population health.

The present study encountered some limitations. We have only used the data of hospitalized patients who were insured by IHIO and not all hospitalization occurred in the study period. Although IHIO is the main financing agent for FPP and HTP claims, but overall inpatient records for entire population should be targeted in future studies. We did not used a controlled ITS design. Although “ITS studies differ from other evaluation designs by making within-group rather than between-group comparisons” [31], but use of a control group with identical demographic and social characteristics that did not experience urban FPP in Iran, would separate the effect of FPP and HTP on hospitalization rate.

## Conclusion

Family physician created a decreasing trend for hospitalization. Development of FPP to urban area of Iran will lead to health system efficiency. HTP with lower user fee in governmental public hospitals and clinics as well as fee-for-services payment mechanism stimulated both level and trend of hospital admissions. More integrated health policy is required to optimize the implementation of diverse simultaneous reforms in low and middle-income countries.

## Data Availability

The datasets used in the study are available from the corresponding author on reasonable request.

## Abbreviations

FPP: Family Physician Plan
HTP: Health Transformation Plan
IHIO: Iran Health Insurance Organization
ITS: Interrupted Time Series
PHC: Primary Health Care
UHC: Universal Health Coverage
